# Susceptibility to poor arguments: The interplay of cognitive sophistication and attitudes

**DOI:** 10.3758/s13421-024-01564-1

**Published:** 2024-04-24

**Authors:** Pinja M. Marin, Marjaana Lindeman, Annika M. Svedholm-Häkkinen

**Affiliations:** 1 Department of Psychology and Logopedics, Faculty of Medicine, University of Helsinki, P.O. Box 21, 00014 Helsinki, Finland; 2 Tampere Institute for Advanced Study, Tampere University, Tampere, Finland

**Keywords:** Thinking styles, Scientific literacy, Motivated reasoning, Attitudes, Argument

## Abstract

Despite everyday argumentation being crucial to human communication and decision-making, the cognitive determinants of argument evaluation are poorly known. This study examined how attitudes and aspects of cognitive sophistication, i.e., thinking styles and scientific literacy, relate to people’s acceptance of poorly justified arguments (e.g., unwarranted appeals to naturalness) on controversial topics (e.g., genetically modified organisms (GMOs)). The participants were more accepting of poorly justified arguments that aligned with their attitudes compared to those that opposed their attitudes, and this was true regardless of one’s thinking styles or level of scientific literacy. Still, most of the examined aspects of cognitive sophistication were also positively related to fallacy detection. The strongest cognitive predictors of correctly recognizing the fallacies were one’s scientific reasoning ability and active open-mindedness. The results thus imply that decreasing misleading attitude effects, and increasing certain aspects of analytic and scientific thinking, could improve argumentation.

**Supplementary Information:**

The online version contains supplementary material available at 10.3758/s13421-024-01564-1.

## Introduction

Understanding the quality of arguments, i.e., justified claims, is an important civic skill and an integral part to making good decisions in everyday life. For instance, having informed opinions rests heavily on one’s ability to correctly evaluate the quality of justifications behind varying claims. In particular, paying attention to evidence quality may make it easier to discuss controversial topics in a constructive manner. This could in turn lessen exaggerations, conflicts, and unfair treatment of people with opposing views. However, correctly assessing argument quality (i.e., knowing how good a reason is at justifying a claim) is easier said than done. British people were misinformed about Brexit with oversimplified slogans and false figures; disinformation about voter fraud in the 2020 US presidential election convinced most Trump supporters that the election was rigged (Pennycook & Rand, 2021a); and globally doubts about the safety and usefulness of COVID-19 vaccines have been intensified with false claims and faulty argumentation techniques.

While there are stringent normative standards of validity (valid or invalid) for formal deduction, the same is not true for informal reasoning. Fallacies are common errors in informal reasoning (e.g., Dowden, n.d.). Whether something is a fallacy depends on content, form, and situation (e.g., Hahn, 2020). For instance, appealing to authority can sometimes be justified, and sometimes not, depending on the situation and authority. Examples of argument fallacies include attacking one’s opponents rather than their arguments: You're a poor hippie, so your claims about climate change are false"; appealing to naturalness: “eating meat is natural, therefore there is no need for humans to lower meat consumption”; and using slippery-slope arguments: “If we accept euthanasia, people would kill their elderly parents once they become troublesome.”

Considering the societal importance of everyday arguments, alarmingly little is known about the factors that lead people to consider an argument as poorly or well justified, i.e., as weak or strong. Specifically, cognitive studies on reasoning have so far not put much focus on everyday argumentation. Consequently, as Hornikx and Hahn (2012) also point out, informal argumentation needs to be more systematically studied in cognitive psychology.

The purpose of this study was thus to analyze the cognitive and attitudinal mechanisms that underlie people's ability to detect fallacies. The fallacies relate to so-called *hot topics*, which are issues that include social, political, and ethical themes, and are thus entangled with people’s values and ideologies (e.g., Sinatra et al., 2014). Examples of these topics include nuclear power, immigration, and euthanasia. Through better understanding why people find poorly justified arguments appealing, the results could help understand and combat the spread of bad arguments in everyday life.

### The role of attitudes

People have been found to favor and distort information in ways that fit their prior views across varying tasks and types of information, from the evaluation of logical syllogisms (e.g., Sá et al., 1999) and political claims (e.g., Ecker et al., 2014) to the creation of reasons for a topic (e.g., Macpherson & Stanovich, 2007). This *myside bias* has also been the focus of many studies investigating everyday argumentation. These studies have found that everyday arguments with claims that align with one’s beliefs, opinions, and attitudes are thought to be better than arguments with claims that oppose them (e.g., Edwards & Smith, 1996; Stanovich & West, 1997, 2008; Taber et al., 2009; Thompson & Evans, 2012; Wolfe & Kurby, 2016).

However, none of these studies have investigated how this tendency to interpret information in a personally favorable way affects the detection of fallacies in particular. We assume that, as documented regarding other types of everyday arguments, people will also evaluate short fallacies in a mysided way. That is, people will favor fallacies with claims that support their attitudes over fallacies with claims that undermine their attitudes.

### The role of cognitive sophistication

Moreover, this study aims to discover whether people’s level of cognitive sophistication also influences their fallacy evaluations. Specifically, two aspects of people’s cognitive sophistication are examined here: thinking styles and scientific literacy. While attitudes likely lead people to mistakenly accept fallacies with attitude-supporting claims, higher cognitive sophistication might show opposite effects, decreasing fallacy acceptance.

#### Thinking styles

Thinking styles refer to habitual ways of thinking in everyday life. People with a high intuitive thinking style like to rely on their intuitive impressions, to make decisions based on their gut feelings, and to follow their heart as a guide for actions (Norris & Epstein, 2011). In turn, analytic thinkers tend to collect information before making up their minds, seek various points of view before drawing conclusions, think extensively about problems before responding, and adjust the degree of strength in their opinions to the degree of available evidence (Stanovich, 2012). A variety of analytic thinking styles exist but, roughly considered, higher analytic thinking style and lower intuitive thinking style indicate higher cognitive sophistication.

Most research examining the relationship between analytic thinking styles and perceptions of argument quality has focused on people’s tendency to engage in and enjoy effortful processing, i.e., their level of need for cognition (Cacioppo & Petty, 1982). These studies have shown that, compared to those low in need for cognition, undergraduates with a high level of need for cognition are more sensitive to argument quality in texts that advocate for a topic such as increasing tuition or taking vitamin K (Cacioppo et al., 1983, 1986; Smith & Petty, 1996). Leary et al. (2017) in turn found that intellectual humility, i.e., easily accepting that one’s views may be wrong, is positively related to appreciating evidence quality in an essay that argued for dental flossing. There is also evidence that dogmatic and categorical thinking are associated with a decreased ability to evaluate arguments in relation to other arguments in dialogues (e.g., Stanovich & West, 1997, 1998).

As a deviation from previous studies, we examine the evaluation of individually presented classic fallacies. However, we still expect our findings to align with the prior literature and analytic thinking styles to increase with the detection of fallacious arguments. Because earlier studies have either examined a single analytic thinking style (e.g., Cacioppo et al., 1983; Leary et al., 2017) or created a composite measure of it (e.g., Stanovich & West, 1997, 1998), it is difficult to know how a particular analytic thinking style is related to argumentation. We will thus investigate four specific analytic thinking styles: (1) *need for cognition* (Cacioppo & Petty, 1982), (2) *intellectual humility* (Leary et al., 2017), (3) *actively open-minded thinking*, particularly one’s willingness to modify views based on the best available evidence (Baron, 2019), and (4) *cognitive reflection*, which is an index of one’s ability to inhibit intuitive but incorrect answers (e.g., Thomson & Oppenheimer, 2016). Examining these four aspects of analytic thinking simultaneously should reveal their relative importance in fallacy detection.

In line with the findings that one’s tendency to think extensively is related to increased detection of argument quality (reviewed in Cacioppo et al., 1996), one’s disposition to rely on fast intuitive impressions has been correlated with a lowered sensitivity to the quality of counterarguments (Svedholm & Lindeman, 2013). Moreover, higher intuitive thinking has been found to make people more likely to accept false information (e.g., Bago et al., 2020)**,** conspiracy ideation (Lobato & Zimmerman, 2019), and bullshit (Pennycook et al., 2015). Based on these findings, the tendency to trust one’s intuitions in decision making can be expected to increase the acceptance of argument fallacies.

#### Scientific literacy

Besides high analytic thinking styles, good scientific literacy is part of cognitive sophistication. We specifically focus on lay people’s scientific reasoning skills since a lack of scientific reasoning seems to be the backbone of many argumentation errors. For instance, using a single case as evidence is hardly reliable, and confidently stating causal relations without any evidence, or even ideas of possible mechanisms, is antithetical to the scientific method. Although we are not aware of any studies examining how one’s scientific reasoning ability relates to correctness in informal argumentation, it has been linked to a lower susceptibility to pseudo- and antiscientific views (Čavojová et al., 2020; Drummond & Fischhoff, 2017a) and to misinformation (Scherer et al., 2021). Moreover, Metz et al. (2018) found that those who have a good understanding of the nature of science tend to agree that scientific justifications (e.g., scientific evidence, scientific consensus) are generally good reasons to “believe something is true.” Consequently, we expect that better scientific reasoning skills, i.e., having a good understanding of the basic concepts and principles that underlie scientific methods (e.g., knowing that correlation does not equal causality and that confounding variables may affect findings), makes it easier to notice argument fallacies.

### Considering attitudes and cognitive characteristics together

Studying both attitudes and aspects of cognitive sophistication (i.e., low intuitive thinking style, high analytic thinking styles, and high scientific reasoning ability) in the same study allows us to contrast their roles in fallacy evaluation, and to test how this type of cognitive sophistication moderates the way attitudes relate to fallacy evaluations. It is currently unclear whether analytic thinking styles and scientific reasoning ability are positively related to fallacy detection regardless of one’s attitudes, i.e., lessens unwanted attitude effects. This is due to a lack of studies investigating this as well as to contradictory results regarding the way analytic thinking and scientific literacy relate to mysided thinking or to reasoning above the effect of prior views.

Some studies have found no relationship between aspects of cognitive sophistication and reasoning when accounting for the effect of attitudes. For example, Macpherson and Stanovich (2007) found that the level of one’s analytic thinking styles, specifically active open-mindedness and need for cognition, were unrelated to the number of arguments generated supporting versus opposing one’s opinions. Very low to non-existent correlations have also been reported between active open-mindedness and mysided evaluation of information (reviewed in Stanovich & Toplak, 2023) as well as between need for cognition and appreciating the strength of counterarguments even when it is not beneficial to one’s opinions (Svedholm & Lindeman, 2013).

Others have discovered that cognitive sophistication exacerbates biases. Need for cognition has been found to increase with mysided evaluation of an experiment (Macpherson & Stanovich, 2007) and with harsher critique towards information that opposes one’s views (Haugtvedt & Petty, 1992). Cognitive reflection has similarly been related to higher mysided agreement with climate change arguments (Pennycook et al., 2023) and to higher mysided interpretation of information regarding climate change and gun control (e.g., Kahan, 2013; Tappin et al., 2021). One’s scientific reasoning ability has also been associated with heightened mysided evaluation of study information (Tappin et al., 2021), while one’s scientific knowledge has been associated with more polarized views on issues (Drummond & Fischoff, 2017b). These findings are in line with the motivated sophistication account,^1^ which suggests that those with higher analytic thinking and scientific literacy are paradoxically the ones that will twist information the most to support their own views (e.g., Tappin et al., 2021).

Still other studies indicate that people high in need for cognition or in aspects of active open-mindedness are the least biased by their prior stances when they evaluate reasoning regarding their occupation (Klaczynski & Lavallee, 2005), climate change (Caddick & Feist, 2022), drinking age, and abortion (Stanovich & West, 2008). Cognitive reflection has also been found to increase with accurate evaluation of fake news despite one’s political alignment (e.g., Ross et al., 2021), and aspects of active open-mindedness appear positively related to the detection of argument strength even when the detection is not favorable to one’s opinions (Stanovich & West, 1997; Svedholm & Lindeman, 2013). Moreover, denial of human-caused climate change has been reported to increase myside bias when analyzing climate change information (Caddick & Feist, 2022). These findings support what has been called the *classical reasoning account*. This account proposes that more analytically and scientifically minded people are better at reasoning no matter their personal views on a topic (e.g., Pennycook et al., 2023).

The little research that has considered people’s prior motivations, cognitive characteristics, and argument quality in the same study mainly shows that cognitive sophistication improves reasoning about arguments over and beyond belief biases. To our knowledge, all these studies have been conducted using the Argument Evaluation Test (AET; Stanovich & West, 1997). In this test, participants are asked to evaluate the quality of counterarguments in relation to previously given arguments. The test is then analyzed in a way that statistically disentangles the degree to which one’s ratings are determined by one’s personal views and the degree to which they are predicted by the actual strength of the arguments. Studies using the AET have found that those with strong analytic thinking styles are generally better at correctly evaluating the strength of counterarguments no matter their own beliefs (Stanovich & West, 1997; 1998; Svedholm & Lindeman, 2013).

Of note, the AET puts weight on people’s ability to avoid mysided reasoning by mainly valuing people’s appreciation of strong arguments that go against their beliefs and/or people’s denial of weak arguments that align with their beliefs. Moreover, the AET involves both poor and well-justified arguments, leaving open the question whether there is motivated sophistication in the evaluation of poorly justified arguments specifically. Our study will add to this research by showing whether more cognitively sophisticated people are more accurate at detecting both fallacies supporting and opposing their attitudes. Because the classical reasoning account has started to receive more support than the motivated sophisticated account (reviewed in Pennycook & Rand, 2021b), we hypothesize that cognitive sophistication increases the detection of attitude-supporting as well as attitude-opposing fallacies but attitude-supporting fallacies the most.

### Objectives

The aim of this study is to investigate what makes people (un)able to correctly recognize argument fallacies on hot topics. The following hypotheses are set:

1. Fallacies are more poorly detected in arguments that align with one’s attitudes than in arguments that contrast them.
2. Intuitive thinking style is associated with a decreased ability to detect fallacious arguments.
3. Analytic thinking style (i.e., need for cognition, intellectual humility, actively open-minded thinking, and cognitive reflection) is associated with an increased ability to detect fallacious arguments.
4. Scientific literacy is associated with an increased ability to detect fallacious arguments.
5. Cognitive sophistication (i.e., low intuitive thinking style, high analytic thinking styles, and good scientific literacy) lessens the biasing effect of attitudes by increasing the detection of fallacies that align with one’s attitudes more than those that oppose one’s attitudes.

In addition, we will explore which types of argument fallacies are the easiest and which are the most difficult to identify.

## Method

### Participants

The participants were 1,325 Finns, aged 18–90+ years (*M* = 40.2, *SD* = 16.1 years). Of them, 56.9% were women, 40.0% men, and 2.6% other. Their full-time occupations were 32.1% studying, 42.9% working, 12.9% retired, and 11.3% other. Their education levels were compulsory school (1.6%), some (4.4%) or completed (14.4%) high school/vocational school, some (30.1%) or completed (40.8%) university/college, some (4.8%) or completed (3.5%) postgraduate work. Most of the participants reported knowing something about argumentation rules and analysis: 21.9% little, 32.1 % something, 18.4% quite much (e.g., taken a university-level course on the topic), 9.7% much (e.g., taken some university-level courses on the topic), and 4.5% very much (e.g., taken many university-level courses on the topic). No familiarity with argumentation rules and analysis was reported by 12.6% of the participants.

The study was conducted online over 1 month from December 2020 to January 2021. We aimed for a sample size of 1,000. Because effects in this type of research tend to be small, we wanted a sample size that is sufficient to detect even small effects (f^2^ ≥ .02) with high statistical power (≥ 85%) at the conventional alpha < .05 significance level. The number of participants exceeded this goal within the planned 1-month answer time, so data collection was ended. Participants were recruited through university and open university mailing lists, a few college mailing lists, religious and non-religious organizations, political parties, a Facebook post that was shown to adult Finns, and Prolific. All participants were told that the study investigates people’s views of societal topics that divide opinions, which kinds of arguments are taken as well-justified versus poorly justified, and different thinking styles. Sharing the study invitation further was also encouraged. The participants recruited via Prolific (*N* = 102) received a small financial remuneration while others were offered a description of their thinking styles as compensation.

### Measures

#### Argument evaluation

The study contained 50 fallacies and 20 strong arguments used as fillers. Fallacy types for the weak arguments were chosen from the literature (e.g., Hahn, 2020; Hinton, 2021), and the arguments were inspired by discussions and popular articles found online (e.g., ProCon.org). We focused on hot topics that allowed forming balanced and diverse arguments on both sides of the issues. The topics, fallacy types, and specific arguments were chosen based on recommendations from a philosopher with expertise in argumentation.

The arguments dealt with legalization of marijuana use, legalization of euthanasia, acceptance of genetic modification in food, need to further restrict immigration to Finland, and increasing the use of nuclear power in the world. For each topic, there were ten fallacies and four strong arguments. Half were in favor of (pro), and half were opposing (con) each topic. An example of a fallacy for the legalization of cannabis is “Cannabis should be legal because then many abusers of harder drugs would start using it instead.”

We used five types of fallacies: ad hominem (i.e., attacking a person instead of their claim), appeal to nature, appeal to ignorance, circular reasoning, and slippery slope (i.e., appealing to unfounded consequences). An example of an appeal to nature fallacy is “The amount of nuclear power in the world should not be increased because it is unnatural to forcefully split atomic nuclei.” All fallacies can be found in the Appendix, Table 5. There was one pro fallacy and one con fallacy of each fallacy type.

The presentation order of the arguments was randomized. First, all arguments were randomized into four blocks. Second, the participants were asked whether their (a) birth month and (b) birthday was even or odd. The order of the blocks was then randomized based on the four possible combinations. Lastly, the order of the particular arguments inside each block was randomized for each group. In this way, four groups were formed that saw the arguments in a unique order. Before each argument block, the participants were instructed to “evaluate the following arguments based on how well they are justified. Do your best to be objective and only think about the quality of the justifications.” To avoid wrong assumptions, they were also told that “whether your answers in a particular topic are systematic will not be analyzed.” All arguments were rated on a 5-point Likert scale (1 *= very poorly justified*, 5 = *very well justified*). Higher values thus indicate poorer recognition of fallacious arguments.

The following mean variables were calculated. *All fallacies*, *pro* and *con issue fallacies* (e.g., *pro euthanasia fallacies, con euthanasia fallacies,* five items each), and *fallacy types* (e.g., *ad hominem, slippery slope,* ten items each). In addition, *aligning fallacies* and *opposing fallacies* were formed based on the evaluation of fallacies that supported and opposed one’s attitudes on the topics. Descriptive statistics for these are shown in Table 1 and described in the *Results* section. The other assessment scales, described below, were shown between the argument blocks except for attitudes, which were asked about in the beginning of the study.

**Table 1:** Spearman correlations between fallacy acceptance and predictors

		Fallacy									
		Cannabis		Euthanasia		GMO		Immigration		Nuclear power	
	All	Pro	Con	Pro	Con	Pro	Con	Pro	Con	Pro	Con
Age	.14	-.05	.16	.07	.06	-.03	.17	.08	-.08	.002	.19
Familiarity	-.15	-.02	-.15	-.10	.03	.02	-.17	-.09	-.08	-.12	-.13
Intuition	**.27**	.05	.18	.13	.12	-.01	**.29**	.14	.11	.14	**.22**
NfC	**-.24**	-.04	**-.21**	-.10	-.06	-.02	**-.22**	-.12	-.11	-.18	**-.21**
CRT	**-.29**	-.04	**-.21**	-.15	-.07	.007	**-.31**	-.11	-.09	-.13	**-.27**
Humility	-.10	.06	-.14	.002	-.07	.01	-.10	-.002	-.11	-.11	-.05
AOT	**-.38**	-.004	**-.32**	-.10	**-.25**	.04	**-.38**	-.09	**-.28**	**-.31**	**-.24**
SRS	**-.39**	-.11	**-.25**	**-.22**	-.08	-.02	**-.41**	-.12	-.16	**-.23**	**-.30**
Attitude^a^		**.38**	**-.60**	**.35**	**-.47**	**.36**	**-.61**	**.35**	**-.53**	.16	**-.50**
*M*	*2.32*	*2.33*	*2.34*	*2.74*	*2.09*	*2.53*	*2.50*	*2.38*	*2.06*	*1.95*	*2.27*
*SD*	*0.37*	*0.66*	*0.74*	*0.71*	*0.70*	*0.62*	*0.78*	*0.68*	*0.73*	*0.64*	*0.68*

*r*s ≥ |.10|: *p* < .001; *r*s ≥ |.08|: *p* < .01; *r*s ≥ |.06|: *p* < .05. Correlations > |.20| are shown in bold font

#### Analytic and intuitive thinking styles

Four aspects of analytic thinking were examined. *Actively open-minded thinking* (α = .66, *M* = 4.23, *SD* = 0.43) was measured with Baron’s (2019) version of the shortened scale, but one psychometrically unsound item of the original 11 items was excluded (Baron, 2019, note 18). The scale consists of items such as “It is OK to ignore evidence against your established beliefs” (1 = *completely disagree*, 5 = *completely agree*). *Intellectual humility* (α = .71, *M* = 3.97, *SD* = 0.51) was assessed with the six-item Intellectual Humility Scale (Leary et al., 2017) and with one additional item measuring the same tendency “I sometimes find a good argument that challenges some of my firmly held beliefs” from Sosu (2013). The items were rated on a scale from *not at all like me* (1) to *very much like me* (5). *Cognitive reflection* was examined with the four-item CRT-2 scale by Thomson and Oppenheimer (2016), and with one reworded item from the original CRT scale: “Sally is making sun tea. Every hour, the concentration of the tea doubles. If it takes 6 hours for the tea to be ready, how long would it take for the tea to reach half of the final concentration?” (Baron et al., 2015). The questions were open-ended, and the number of correct answers to the five items was used as an index of cognitive reflection for those that had answered at least one item (*M* = 3.20, *SD* = 1.26, α = .57).

*Need for cognition* (α = .77, *M* = 3.80, *SD* = 0.69) and *Faith in intuition* (α = .71, *M* = 2.94, *SD* = 0.69) were measured with five items each, consisting of the items in the REIm-13 (McGuiness et al., 2019) as well as of one additional item measuring Need for Cognition (“I prefer complex to simple problems“) and of two items measuring intuitive thinking (“I like to rely on my intuitive impressions“ and “I often go by my instincts when deciding on a course of action“). The added items were taken from the original REIm scale and correlated highly with the items in the shortened version (Norris & Epstein, 2011). They were included to ensure reliability. All items were answered on a 5-point Likert scale (1 = *strongly disagree*, 5 = *strongly agree*).

#### Scientific literacy

The participants’ scientific literacy was assessed with eight (out of the original 11) problems from the Scientific Reasoning Scale (Drummond & Fischhoff, 2017a). Each problem consists of a short scientific scenario that is followed by a conclusion. For instance, understanding of causality is measured with “A researcher finds that American states with larger parks have fewer endangered species. These data show that increasing the size of American state parks will reduce the number of endangered species.” Following Čavojová et al. (2020), the answer options to all statements were *true, false,* and *I don’t know*. The level of scientific reasoning skills was calculated as the number of correct answers for those that had answered at least two items (*M* = 6.03, *SD* = 1.63). The included items asked about blinding, causality, confounding variables, control group, ecological validity, history, maturation, and random assignment (α = .55).

#### Attitudes

*Attitudes towards the hot topics* were investigated with three pairs of adjectives: *bad – good*, *foolish – wise*, and *harmful – beneficial*. Each pair was rated on a 7-point scale, in which the adjectives formed the end labels and the middle option (4) was labeled “in between.” Higher values denote a more positive view of an issue. Each topic had a similar instruction with only the issue varying. For instance, participants were asked: “What is your opinion on legalizing euthanasia? Legalizing euthanasia is…”. The five attitude scales had good reliabilities (α = .95 - .97) and similar standard deviations (1.53–1.84). Also, their mean values were quite equal and varied from 4.09 (cannabis) to 5.32 (euthanasia). The participants’ attitude toward a topic was calculated if at least two of the adjective pairs were answered.

Participants whose attitude toward a topic was above four were coded as taking the pro stance on a topic and participants with values less than four were coded as taking the con stance. These were used to code fallacies as aligning and opposing for each participant individually. For instance, aligning arguments would be the pro arguments for those with a pro stance and the con arguments for those with a con stance on a specific topic. The mean variables of *aligning and opposing fallacies* were formed for the participants that had either a positive (> 4) or a negative (< 4) attitude towards at least four of the five topics. For those with more than one neutral or missing attitude, these variables were coded as missing.

#### Composite variables

If not otherwise stated, the composite variables were computed as a mean of answers as long as a participant had less than 25% of answers missing on a particular scale. The *aligning fallacies* and *opposing fallacies* were missing from 13% of the participants, and one or more of the other composite variables was missing from 3% of the participants. Skewness was greater than |1| for two of the variables: *con immigration fallacies* (1.04), and *attitude towards euthanasia* (-1.03). Multicollinearity between the predictors was acceptable in all upcoming regression analyses (Tolerance > 0.5 and VIF < 1.7), and the effect size estimates provided by Cohen (1992) were used to interpret the results, i.e., *R*^2^ > .02 was considered a small, *R*^2^ > .13 moderate, and *R*^2^ > 0.26 a large effect.

## Results

First, a repeated-measures ANOVA was done to contrast the means of aligning (*M* = 2.63, *SD* = 0.55) and opposing (*M* = 1.98, *SD* = 0.41) fallacies. Supporting Hypothesis 1, poorly justified arguments that supported one’s attitudes were perceived as more justified than those that opposed one’s attitudes,* F*(1,1155) = 1295.15, *p* < .001. Second, the Friedman test was conducted to compare the fallacy types with each other, *F*_*r*_ (4) = 2367.49, *p* < .001. Except for a lack of difference between the ratings of ignorance and slippery slope arguments (*p* = .074 after Bonferroni correction), the evaluation of other fallacy types differed from each other (Bonferroni corrected *p*s < .001). The fallacies from easiest to most difficult to detect were: ad hominem (*M* = 1.71, *SD* = 0.59), appeal to nature (*M* = 2.19, *SD* = 0.55), circular reasoning (*M* = 2.41, *SD* = 0.45), and last appeal to ignorance (*M* = 2.61, *SD* = 0.47) together with slippery-slope arguments (*M* = 2.67, *SD* = 0.52). Acceptance of the five fallacy types correlated positively with each other (*r*s [.25, .56], Table S1 in the Online Supplementary Material (OSM)), and aspects of cognitive sophistication were most robustly negatively correlated with the acceptance of ad hominem and appeal to natural fallacies (Table S2, OSM). For more specific information regarding the fallacy types, see Tables S1 and S2 (OSM).

Next, we examined correlations between the predictors and the overall as well as topic specific fallacy evaluations (Table 1). These showed that the participants evaluated the poor arguments in line with their attitudes on every topic, being more lenient towards aligning than opposing fallacies. Attitudes also appeared to be more strongly related to the evaluation of the con topic fallacies than the pro topic fallacies. Regarding the cognitive characteristics, intuitive thinking style increased while aspects of analytic thinking and scientific literacy decreased with the overall level of fallacy acceptance. These results support Hypotheses 2–4, although support for Hypothesis 3 regarding intellectual humility was inconclusive since it decreased with fallacy acceptance very weakly. Moreover, the direction of the correlations was quite uniform across the topics although the strength of these correlations varied. Correlations between the cognitive characteristics ranged from nonexistent to moderate (*r*s [|.05|,|.38|], Table S2 (OSM)).

To get a clearer view of how consequential the cognitive characteristics were in fallacy detection, we performed a stepwise regression analysis predicting the overall level of fallacy acceptance (Table 2). The results showed that after adjusting for age and familiarity with argumentation, one’s thinking styles and scientific literacy explained a moderate amount of variation (21%) in the way the fallacies were perceived. Actively open-minded thinking and scientific reasoning skills mattered the most in correctly rating the fallacies. In contrast, need for cognition was not related to fallacy evaluation once age, familiarity with argumentation, and the other cognitive characteristics were considered. The regression analysis thus also supports Hypotheses 2 and 4 but only supports Hypothesis 3 regarding active open-mindedness and cognitive reflection. We left intellectual humility out of the regression model for clarity (It had a weak association with fallacy detection to start with [Table 1], and including intellectual humility into the model changed its relationship to be weakly positive with fallacy acceptance [β = .07, p = .02] but did not change conclusions regarding the other cognitive characteristics).

**Table 2:** Results of hierarchical regression analysis predicting the overall level of fallacy acceptance

	All fallacies	
	β	CI (95%)
Block 1: Background		
Age	.16***	[.10, .21]
Familiarity	-.14***	[-.20, -.09]
*Adj. R*^*2*^	*4.5 %*	
Block 2: Cognition		
Age	.04	[-.01, .09]
Familiarity	-.03	[-.08, .02]
Intuition	.14***	[.09, .19]
Need for cognition	-.05	[-.10, .00]
Cognitive reflection	-.10***	[-.15, -.05]
Active open-mindedness	-.22***	[-.27, -.16]
Scientific reasoning skills	-.23***	[-.29, -.18]
*Δ Adj. R*^*2*^	*21.2 %*	

***p < .001; **p < .01; *p < .05

Familiarity = familiarity with argumentation

Changes in Adj. *R*^2^ after each block were significant at *p* < .001

### Separating the roles of cognition and attitudes

To compare the roles that attitudes and the cognitive characteristics have in fallacy detection, we ran ten separate hierarchical regression analyses: one for each side of a topic. The first block of predictors included the background variables, the second block attitudes, and the third block cognitive sophistication and its interaction with attitudes. The interactions were included to test whether the cognitive characteristics predict noticing fallacies that support versus oppose one’s attitudes differently. For instance, scientific reasoning ability and analytic thinking styles could be negatively correlated with the acceptance of con topic fallacies in particular (Table 1) because participants higher in these aspects of cognitive sophistication tended to have positive attitudes towards the examined topics (*r*s [.01, .35], Table S2 (OSM)).

To simplify the analyses, we used a cognitive sophistication composite variable (*M* = 0, *SD* = 0.67, range = [-2.47, 1.56]) consisting of those characteristics that were related to fallacy detection (*p* < .05) in the regression analysis predicting overall fallacy acceptance (Table 2). The (standardized) variables included were: intuitive thinking style (reversed), cognitive reflection, actively open-minded thinking, and scientific reasoning skills. Additional analyses in which all cognitive characteristics and their interactions with attitudes were entered individually in block 3 are shown in Tables S3–S4. These analyses with all variables included did not change the conclusions and revealed overlap between the cognitive characteristics in predicting the fallacies independently of attitude, thus supporting the use of the composite measure.

Table 3 shows the results of the topic-specific analyses (confidence intervals in Table S5 (OSM)). To correct against type 1 error, we applied a Bonferroni-corrected significance level of *p* < .005 (.05/10). Self-evaluated familiarity with argumentation had an independent effect on fallacy detection only in a couple of instances and even these effects were small. Attitudes and cognitive sophistication in turn showed clearly unique roles in fallacy acceptance. However, on average, attitudes toward a given topic covered notably more of the variation in fallacy evaluations (a moderate amount, 21.0%) than thinking styles and scientific literacy combined (which explained only a small amount, 7.4%). Cognitive sophistication weakly improved all other fallacy evaluations except for the detection of *pro nuclear fallacies*, which it improved moderately. Attitudes, in turn, had only a small role in the *pro nuclear fallacy* ratings, but otherwise were a medium to large predictor of how the fallacies were perceived. The results also showed that attitudes were moderately related to accepting the pro topic fallacies (13.2%) and strongly associated with accepting the con topic fallacies (28.8%). The more one’s attitudes aligned with the fallacy claims, the more likely one was thus to accept the fallacies, but this tendency appeared to be stronger for the con topic than pro topic fallacies.

**Table 3:** Betas of hierarchical regression analyses predicting the acceptance of pro and con topic fallacious arguments

	Cannabis+	Cannabis-	Euthanasia+	Euthanasia-	GMO+	GMO-	Immigration+	Immigration-	Nuclear+	Nuclear-
Block 1: Background										
Age	-.05	.21***	.09**	.08**	-.04	.20***	.10***	-.03	.04	.22***
Familiarity	-.02	-.14***	-.10***	.04	.02	-.14***	-.08**	-.09***	-.10***	-.11***
*Adj. R*^*2*^	*0.2 %*	*6.4 %*	*1.7 %*	*0.6 %*	*<.01 %*	*6.0 %*	*1.4 %*	*0.8 %*	*1.1 %*	*5.9 %*
Block 2: Attitude										
Age	.04	.08***	.12***	.04	.09***	.01	.05*	.03	.09**	.11***
Familiarity	-.07**	-.07**	-.07**	-.01	-.02	-.09***	-.13***	-.02	-.12***	-.08***
Attitude^a^	.41***	.59***	.41***	.52***	.43***	.61***	.38***	.56***	.21***	.47***
*Δ Adj. R*^*2*^	*15.5 %*	*32.0 %*	*16.5 %*	*26.7 %*	*16.2 %*	*33.5 %*	*14.1 %*	*30.9 %*	*3.9 %*	*21.1 %*
Block 3: Cognition										
Age	.01	.03	.04	.01	.07**	-.03	-.01	-.04	<.01	.06*
Familiarity	-.03	-.05*	<.01	.01	.01	-.05*	-.09***	.02	-.05	-.03
Attitude^a^	.46***	.52***	.42***	.47***	.50***	.49***	.41***	.51***	.30***	.40***
Cognition	-.25***	-.18***	-.29***	-.14***	-.22***	-.28***	-.22***	-.20***	-.40***	-.28***
Attitude*Cognition	-.17***	-.14***	-.14***	-.15***	-.12***	-.05**	-.12***	-.20***	-.18***	-.15***
*Δ Adj. R*^*2*^	*7.2 %*	*4.6 %*	*9.3 %*	*4.0 %*	*4.4 %*	*6.2 %*	*4.5 %*	*8.6 %*	*16.4 %*	*8.7 %*

***p < .001; **p < .01; *p < .05. Betas significant at *p* < .05 after Bonferroni correction are shown in bold font

**Fig. 1:**
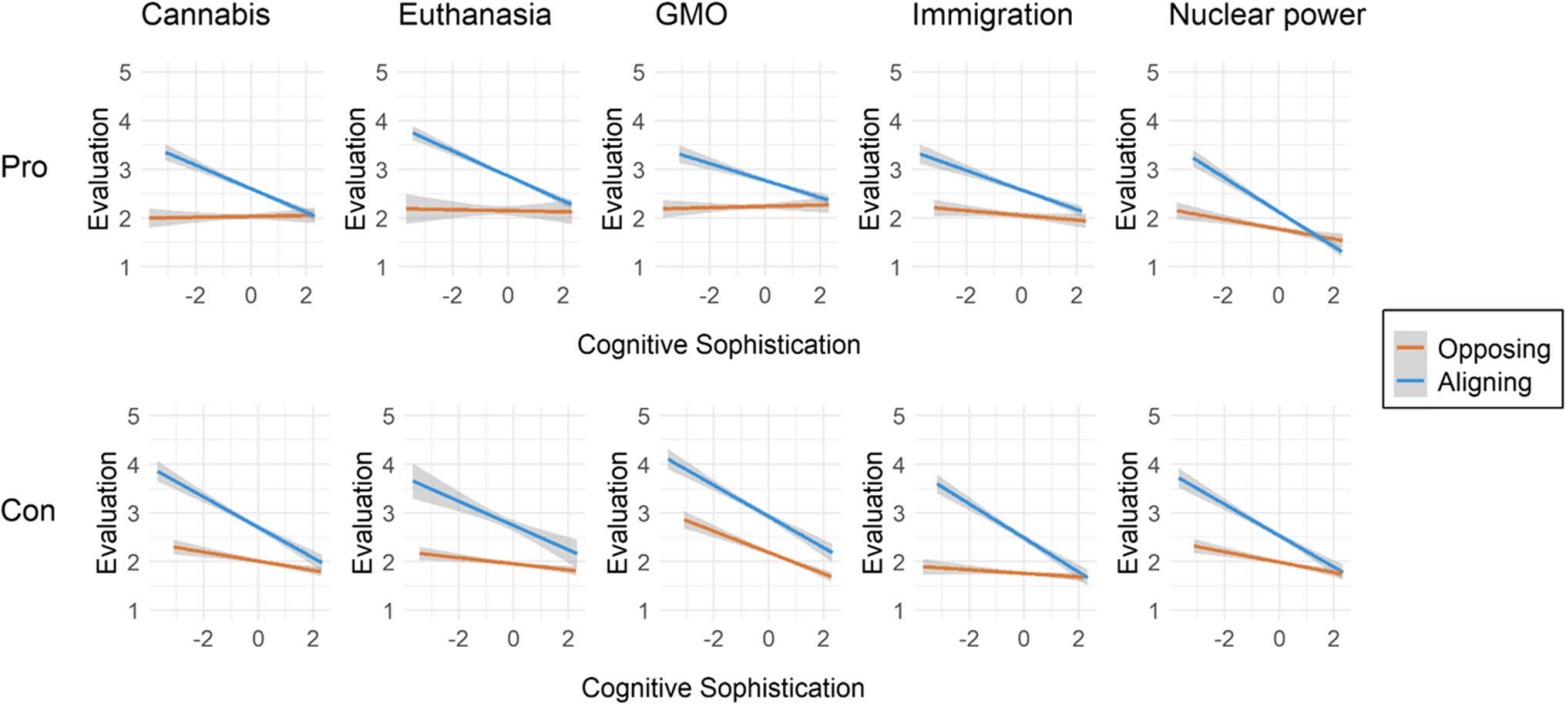
Correlations between fallacy acceptance and cognitive sophistication, calculated separately for participants whose attitudes aligned with and opposed the fallacy claims

The interactions between cognitive sophistication and attitudes were also significant. To understand the direction of these interactions, we calculated the correlations between cognitive sophistication and the evaluation of pro and con topic fallacies separately among participants with a pro stance and those with a con stance. These results support Hypothesis 5 by showing that cognitive sophistication was more consequential to correctly rating the fallacies when their claims aligned with one’s attitudes than when they opposed one’s attitudes (Fig. 1). Cognitive sophistication correlated on average moderately with the acceptance of fallacies that aligned with one’s attitudes (*r* = -.39, range = [-.49, -.25], *p*s < .001) while its correlation with opposing fallacies was weak (r = -.11, range= [-.32, .02], *r*s >|.09|: *p* < .05, *r*s >|.10|: *p* < .001). This variation is understandable because detecting fallacies that oppose one’s attitude takes little cognitive effort and leaves little room for the cognitive characteristics to improve performance, leading to a floor effect. Two additional hierarchical regressions confirmed that the role of cognitive characteristics in correctly rating the fallacies was moderate when the fallacy claims aligned with one’s attitudes but only small when the fallacy claims opposed one’s attitudes (Table 4). In sum, attitudes had a larger effect than the cognitive characteristics in fallacy evaluations but cognitive sophistication notably increased fallacy detection when it was needed to arrive at the correct answer.

The findings further suggested some differences between the cognitive characteristics in how they predicted aligning versus opposing fallacies (Table 4). In particular, active open-mindedness appeared to more evenly decrease the acceptance of fallacies supporting and opposing one’s attitudes compared to cognitive reflection or scientific reasoning ability, which mainly predicted noticing attitude-supporting fallacies. Moreover, need for cognition only lowered the acceptance of fallacies that opposed one’s attitudes.

**Table 4:** Results of hierarchical regression analyses predicting the acceptance of fallacies aligning with and opposing one’s attitudes separately

	Aligning fallacies		Opposing fallacies	
	Β	CI (95%)	β	CI (95%)
Block 1: Background				
Age	.25***	[.19, .31]	-.06	[-.11, .003]
Familiarity	-.12***	[-.17, -.06]	-.10***	[-.16, -.04]
*Adj. R*^*2*^	*7.6 %*		*1.1 %*	
Block 2: Cognition				
Age	.13***	[.08, .18]	-.08*	[-.14, -.02]
Familiarity	-.03	[-.08, .03]	-.03	[-.09, .03]
Intuition	.16***	[.11, .21]	.07*	[.01, .13]
Need for cognition	<.01	[-.06, .06]	-.10**	[-.16, -.03]
Cognitive reflection	-.13***	[-.19, -.08]	.04	[-.02, .11]
Intellectual humility	.05	[-.002, .11]	.05	[-.01, .12]
Active open-mindedness	-.18***	[-.24, -.12]	-.17***	[-.24, -.10]
Scientific reasoning skills	-.26***	[-.32, -.21]	-.08*	[-.15, -.02]
*Δ Adj. R*^*2*^	*21.5 %*		*5.7 %*	

***p < .001, **p < .01, * p < .05

*Familiarity *= familiarity with argumentation

## Discussion

Our goal was to analyze how attitudes and cognitive characteristics relate to the way people evaluate poorly justified informal arguments. The results showed that across the five hot topics, ranging from cannabis legalization and genetically modified organism (GMO) acceptance to decisions regarding euthanasia, immigration, and nuclear power, people’s attitudes towards a topic were more consequential than their cognitive characteristics in predicting how poor or well justified the fallacies were considered to be. More specifically, attitudes were more consequential than the cognitive characteristics in nine out of ten fallacy set evaluations, explaining at least a moderate amount of variation in the way people perceived the hot topic fallacies. This said, aspects of cognitive sophistication were not meaningless, and their effect became clear on fallacies on which correct responding required rejecting an argument for a personally held view.

### Attitudes in fallacy detection

The clear role that attitudes had in people’s fallacy evaluations supports our first hypothesis: fallacies that supported one’s attitudes were harder to view as poor compared to fallacies that opposed one’s attitudes. For example, people who considered that legalizing cannabis is a good thing favored poorly justified arguments that argued for its legalization (“because those who oppose it create unnecessary horror scenarios of mild drugs”) over those that described why it should not be legalized (“because those who support its legalization have too rosy a view of drugs”).

These findings add to the literature by showing that people are notably biased by their attitudes also when they evaluated short poorly justified arguments that consist of fallacies. Moreover, attitudes were most consequential when people considered fallacies opposing an issue, perhaps because the negation in these fallacies made them less obviously weak, and thus easier to accept, compared to the fallacies supporting an issue (e.g., GMOs should not be allowed because people should leave natural cycles alone, vs. GMOs should be allowed because genetic mutations happen in nature anyway). One’s attitudes thus seem to color the assessment of various kinds of arguments (e.g., Taber et al., 2009; Thompson & Evans, 2012), but those arguing against a topic possibly more than those arguing for it.

We also add to previous findings by directly comparing how consequential one’s attitudes versus cognitive characteristics are in evaluating the quality of poorly justified arguments. The results showed that in all five hot topics, attitudes affected the fallacy evaluations even when taking into account thinking styles and scientific literacy. This study thus extends Stanovich and West’s (2008) finding that attitude biases in argumentation exist regardless of one’s intelligence level. It appears difficult for people to completely escape their attitudes, no matter their level of scientific literacy, analytic thinking, or intelligence. Perhaps gently discussing reasoning behind people’s own and others’ views would be useful to everyone, no matter if one is more analytically minded or holds a more evidence-based view of the topic in question.

### Cognitive sophistication in fallacy detection

The correlations that thinking styles and scientific literacy had with the overall level of fallacy detection supported our next three hypotheses: higher analytic thinking styles and scientific reasoning ability, and lower reliance on intuitive feelings in everyday situations, predicted better ability to identify poorly justified arguments. These results align with and broaden previous findings that intuitive thinking style is negatively related (Svedholm & Lindeman, 2013), while need for cognition (Cacioppo et al., 1983; 1986; Smith & Petty, 1996), intellectual humility (Leary et al., 2017), aspects of actively open-minded thinking (Stanovich & West, 1997), and cognitive ability (Stanovich & West 1997; 1998; Eagly & Warren, 1976) are positively related to detecting the quality of informal argumentation. The findings also match reports that analytic thinking increases with one’s likelihood to base their attitudes on solid information (Cacioppo et al., 1983, 1986; Eagly & Warren, 1976) and to provide evidence-based justifications for their view (Sá et al., 2005; Lobato & Zimmerman, 2019).

Taken together, there is accumulating evidence that the higher one’s analytic thinking and scientific literacy, the better the quality of justifications one requires. Moreover, this tendency appears to span across different arguments, from the detection of short logical fallacies to sensitivity to evidence quality in dialogues (Stanovich & West, 1997) and in longer texts (Cacioppo et al., 1996; Leary et al., 2017). Since people with different levels of cognitive sophistication are known to differ in their level of skepticism towards various unwarranted claims, such as fake news (e.g., Ross et al., 2021), vague statements (e.g., Pennycook et al., 2015), and unscientific information (e.g., Fasce & Picó, 2019), these differences could be due to their different demand for evidence quality. Cognitive sophistication may thus make it easier to accept evidence-based views due to making people skeptical of poor reasoning but convinced by well-justified argumentation.

### Motivated sophistication in fallacy detection

In line with our fifth hypothesis, we found no evidence of motivated sophistication. Cognitive sophistication, i.e., cognitive reflection, active open-mindedness, scientific reasoning ability, and low faith in intuition, had an effect over and beyond attitudes: It was related to better fallacy detection whether the fallacies supported or opposed one’s attitudes. At the same time, its effect was moderated by attitudes: Cognitive sophistication particularly helped in dismissing the attitude-supporting fallacies. This is understandable since noticing problems in fallacies that oppose one’s attitudes requires one to simply think in line with one’s attitudes. Since scientific reasoning and analytic thinking appeared overall more negatively related to the acceptance of attitude-supporting than attitude-opposing fallacies, those higher in cognitive sophistication also displayed less mysided evaluation of the fallacies. Moreover, none of the examined aspects of cognitive sophistication increased with fallacy acceptance even when the fallacies aligned with one’s attitudes.

These findings extend previous results indicating that intelligence and analytic thinking lowers unreasonable mysided thinking by helping people to disengage from their own views when they would lead to the incorrect answer regarding argument quality (Stanovich & West, 1997, 1998). Together with these studies, the present results support the view that reasoning tendencies help (as the Classical Reasoning account states; Ross et al., 2021) over that they hurt (as the Motivated Sophistication account states; Kahan, 2015; Stagnaro et al., 2023) people in making rational decisions on a personally significant topic. At the same time, the findings highlight two issues that make contrasting the broad motivated sophistication and classical reasoning accounts problematic. Paying attention to these issues should help clarify how cognitive sophistication relates to motivated reasoning.

First, various types of mysided thinking exists. We propose that considering the correctness of answers can help understand some of the seemingly conflicting findings because sometimes reasoning in line with a given view is justified, not a sign of bias (e.g., Thompson & Evans, 2012). For instance, agreeing the most with climate change arguments that align with one’s presumed views is justified if one holds an evidence-based view of the matter (Pennycook et al., 2023). While higher cognitive sophistication has been connected to larger polarization (e.g., Kahan et al., 2017; Pennycook et al., 2020; Shoots-Reinhard et al., 2021), to increased counter-argumentation (Eagly & Warren, 1976; Haugtvedt & Petty, 1992), and to being more affected by one’s position when interpreting information (e.g., Baker et al., 2020; Kahan, 2013; Tappin et al., 2021), only little evidence exists that cognitive sophistication would lead away from accuracy or increase irrational biases (but see Kahan, 2015; Pennycook et al., 2023; Ross et al., 2021). Cognitive sophistication does thus sometimes increase thinking in line with one’s views, but not generally at the expense of the correctness of this thinking.

### Individual aspects of cognitive sophistication

Second, cognitive sophistication is a diverse concept and different types of intelligence measures (Shoots-Reinhard et al., 2021; Stanovich & West, 1998), science literacy indexes (Drummond & Fischoff, 2017b; Kahan, 2015), and analytic thinking composites (Pennycook et al., 2023; Stanovich & West, 1998) have been used to approximate it. This variability makes drawing conclusions difficult because, as this and previous studies (e.g., Drummond & Fischoff, 2017b Shoots-Reinhard et al., 2021; Svedholm & Lindeman, 2013) show, not all aspects of cognitive sophistication relate to reasoning similarly. While none of the cognitive characteristics that we assumed to reflect higher cognitive sophistication hindered fallacy detection, some were unrelated to it and some lessened mysided thinking more than others.

Deviating from our second hypothesis, one’s willingness to ponder and carefully justify views did not predict the overall fallacy evaluations when other cognitive characteristics were accounted for. The positive association between need for cognition and detection of argument quality in this and prior research (reviewed in Cacioppo et al., 1996) could thus at least partly be due to deliberation being related to other aspects of cognitive sophistication. Moreover, need for cognition did not increase with one’s ability to notice fallacies aligning with one’s attitudes. This matches Svedholm and Lindeman’s (2013) finding that need for cognition is unrelated to people’s ability to correctly evaluate counterarguments above the effect of their beliefs. The lack of a clear association between intellectual humility and better fallacy detection in the present study was also unexpected. However, while Leary et al. (2017) did find that those with higher intellectual humility are more sensitive to argument quality in an essay that supported flossing, this finding did not extend to those who flossed. Being generally humble regarding own views or merely thinking about something does therefore not appear to help people to detect the quality of various types of information.

In contrast, intuitive thinking style and cognitive reflection did predict fallacy evaluation as expected: The disposition to avoid intuition in everyday life and the tendency to resist intuitively tempting but incorrect answers weakly increased with correctly evaluating the fallacies. These findings add to research showing that lower intuitive thinking style and higher cognitive reflection are associated with skepticism towards meaningless sentences (e.g., Pennycook et al., 2015). Moreover, the present analyses indicated that low reliance on intuitive processing was more associated with correct detection of fallacies that supported one’s attitudes compared to fallacies that opposed one’s attitudes, i.e. lowered mysided reasoning. This fits the positive association between intuitive thinking style and mistakenly judging the strength of counterarguments based on their alignment with one’s beliefs, not their objective strength (Svedholm & Lindeman, 2013).

Lastly, understanding scientific information and being actively open-minded appeared to help in evaluating argument quality regardless of the topic in question. These findings support Sagan’s (1997, p. 196) suggestion that through learning scientific reasoning, scientists have a “baloney detection kit” at their disposal, which helps them think skeptically and separate between well-reasoned and fallacious arguments. The results also extend the previously found positive correlation between aspects of active open-mindedness and one’s sensitivity to argument quality in dialogues (e.g., Stanovich & West, 1997). This heightened sensitivity to evidence quality among scientifically literate and actively open-minded people could explain why they are particularly likely to hold evidence-based views, for example, accept evolution (e.g., Metz et al., 2018; Pennycook et al., 2020).

Although active open-mindedness and scientific reasoning skills both strongly increased with the overall fallacy detection, they appeared to relate to mysided thinking differently. Actively open-minded thinking decreased the acceptance of fallacies opposing and aligning with one’s attitudes evenly while scientific reasoning skills appeared to mainly help noticing problems in fallacies that aligned with one’s attitudes. Being good at scientific reasoning could thus protect people from automatically thinking in line with their attitudes, making people particularly open to valid thinking that goes contrary to their own views. On the other hand, actively open-minded thinking may help people notice mistakes (or valid points) in reasoning similarly whether they support or oppose one’s attitudes. If this is the case, then active open-mindedness would not show any relationship to mysided evaluation of incorrect (or correct) information since it is calculated as acceptance of information supporting one’s views minus acceptance of information opposing them. This could explain why previous studies have failed to find a relationship between one’s level of active open-mindedness and myside bias (Stanovich & Toplak, 2023). This tendency of actively open-minded thinkers to dismiss poorly justified statements regardless of their attitudes may reflect their high motivation toward accuracy (Kunda, 1990).

One’s ability to evaluate scientific evidence and tendency to critically reflect on intuitions thus appeared to lower mysided thinking while active open-mindedness was only related to the correctness of thinking, and intellectual humility was unrelated to both. That both scientific reasoning skills and cognitive reflection lessened mysided thinking in this study could be due to their overlap with one’s cognitive ability. At the same time, the present differences between analytic thinking styles strengthen previous findings that even conceptually close measures of cognitive sophistication can differ (e.g., Fasce & Picó, 2019; Klaczynski, 1997; Pennycook et al., 2020). This highlights the importance of being specific about the way cognitive sophistication is examined and creating composite measures of cognitive sophistication only from aspects that behave similarly in relation to the outcome.

### Differences between the fallacy types

This study also allowed examining different fallacy types. The five examined fallacies appeared to capture a common phenomenon of questionable justifications as they were all positively related to each other (Table S1 (OSM)). At the same time, some fallacy types were easier to notice than others. Ad hominem fallacies were notably the easiest to recognize as weak, followed by natural fallacies and then by circular arguments. Arguments that appealed to ignorance or included slippery slopes were the most difficult to identify as weak. However, this order was dependent on whether the pro or con form of the fallacy types were examined with only the ad hominem arguments being systematically the easiest to detect (Table S1 (OSM)). Students have also viewed that attacking a person rather than their claim is less acceptable argumentation than concluding that a statement is true because there is no evidence against it (Neuman et al., 2006). Moreover, while higher cognitive sophistication never clearly increased with fallacy acceptance, it was most robustly negatively related to the ad hominem and natural fallacies (Table S2 (OSM)).

These differences in the detection of specific fallacy types as well as in the way they correlated with the cognitive characteristics and with each other, may be due to their nature: attacking a person and appealing to nature were likely more consistent across various topics and topic sides, while circular reasoning, slippery slopes, and appeals to ignorance were more form and content dependent, making them harder to recognize due to a lack of a simple characteristic. As Hahn and Oaksford (2006) point out, the strength of an appeal to ignorance depends on the amount of possible research conducted and the soundness of a slippery slope varies based on the probability of the consequence. Differences in the endorsement of various fallacy types could also be caused by differences in their persuasiveness, not just in their level of obviously poor reasoning.

While more research is needed to better understand differences and similarities between distinct fallacies, this study suggests that investigating specific types of weak and strong arguments may be one way to enhance our understanding of informal argumentation. Overall, the findings encourage further investigations into the way the structure and objective strength of arguments affects people’s reasoning on a particular topic. Paying closer attention to details, such as argument types and their alignment with one’s attitudes, should also help combine findings from varied approaches to human argumentation (e.g., Eagly, 1999; Klaczynski, 1997; Lobato & Zimmerman, 2019; Sá et al., 2005; Smith & Petty, 1996; Stanovich & West, 1997; Taber et al., 2009).

### Limitations

The present results are restricted to the chosen fallacies. Future studies could, for instance, investigate whether cognitive sophistication similarly increases one’s ability to detect weak and strong argumentation (Bago et al., 2020; Cacioppo et al., 1983). The arguments’ objective strength could also be better defined. In the present materials, the fallacies that supported nuclear power were, for some reason, not very tempting for those supporting nuclear power. More research is needed to understand why. Some of the fallacies should also be made more clearly weak. For instance, stating that GMOs *may have* unknown health risks is less dependent on knowledge and more clearly weak than saying that GMO’s health risks *are* unknown. Moreover, this study did not assess participants’ prior knowledge, which may have affected reasoning particularly on some of the fallacies. Future research could disentangle the effects of prior knowledge by measuring it.

The studied topics may also affect the findings since cognitive sophistication may increase questionable mysided thinking only on ambiguous enough topics with specific type of contestation. Particularly complex and divisive issues, such as climate change risks and action, seem necessary to elicit motivated sophistication across the political spectrum (e.g., Drummond & Fischoff, 2017b; Kahan, 2015; Pennycook et al., 2023). Overall, cognitive sophistication has been found to increase mysided interpretation of information regarding gun control, climate change, and COVID-19 (e.g., Chung et al., 2023; Kahan et al., 2017 Shoots-Reinhard, 2021; Tappin et al., 2021), but not other topics (e.g., this study; Stagnaro et al., 2023; Stanovich & West, 1997). Because the topics of this study were more endorsed by cognitively sophisticated individuals (*r*s [.11, .46], Table S2 (OSM)), and likely more supported by liberals than conservatives, other types of topics remain to be investigated. Future studies could also consider people’s attitudes towards topics (e.g., their valence, extremity, and evidence-base) and investigate if it would explain why those with higher cognitive sophistication are more affected by their views when they interpret politically charged information as well as why cognitive sophistication increases with mysided thinking more among some than others (Baker et al., 2020; Kahan, 2013; Pennycook et al., 2023; Ross et al., 2021; Tappin et al., 2021).

That we did not measure intelligence can be viewed as a limit since it has been shown to be related to argument evaluation (Klaczynski, 1997; Stanovich & West, 1997), mysided thinking (Shoots-Reinhard et al., 2021; Tappin et al., 2021), and the examined cognitive characteristics (Drummond & Fischoff, 2017a; Pennycook et al., 2015; Stanovich & West, 1997). Measuring verbal and numerical cognitive ability together with thinking styles and scientific reasoning skills is therefore necessary to understanding their independent roles in human argumentation, and in motivated reasoning more generally. This could be particularly important for measures with clear overlap with one’s ability. Interactions could also exist between thinking styles and cognitive ability in relation to argumentation (Ståhl & van Prooijen, 2018). For instance, high active open-mindedness may only be helpful in arguing fairly and correctly among those with sufficient intelligence. Intelligence could in turn increase unreasonable motivated reasoning when one lacks sufficient motivation to be rational. It is thus possible that without intrinsic or situational motivation to be rational, cognitive ability would behave contrary to many analytic thinking styles and hinder reasoning on issues central to oneself (e.g., Chung et al., 2023; Stanovich, 2012).

Overall, the study situation may need to be open enough for motivated sophistication to appear. This study characterizes how people evaluate arguments in an objective situation in which participants are told to “be as impartial as possible when evaluating the quality of the justifications.” More indirect cues to be impartial also existed since the participants were asked about their attitudes beforehand and saw both opposing and supporting arguments. This differs from studies finding motivated sophistication which have not asked participants to ignore their own views when interpreting information and have made the variables of interest less clear (e.g., Kahan, 2013; Pennycook et al., 2023; Ross et al., 2021; Tappin et al., 2021). Thus, it is possible that if rationality cues were absent, the same cognitive characteristics that we, and studies using the AET (e.g., Stanovich & West, 1997; 1998), found helpful might instead increase questionable motivated reasoning. While this might be most likely regarding cognitive ability, information about it is necessary to test our findings. On the other hand, one’s tendency to deliberate (as well as other analytic thinking styles) could be more helpful when accuracy and even-handedness are not emphasized, or even cued, and less cognitive effort is demanded (e.g., Cacioppo et al., 1986; Leary et al., 2017).

Lastly, this study is bound by the used sample, which was quite analytic and educated. The role of cognition and attitudes in fallacy detection remain therefore to be investigated among more varied individuals. The participants in this study were also not very susceptible to the fallacious arguments with most of the participants considering them to be poorly justified. Since our results imply that less analytic people and those lower in scientific literacy are most likely to accept poor arguments, cognitive characteristics could come out as more critical to argument evaluation among a more heterogeneous sample than what was found here. Similar studies in different cultures are also needed to see how our findings generalize. For instance, nearly all evidence of motivated sophistication comes from the USA.

### Conclusions

This study contrasted the roles of attitudes versus cognitive sophistication in fallacy detection. People were clearly affected by their attitudes when evaluating the fallacies: fallacies that opposed one’s attitudes were judged more critically than fallacies that supported one’s attitudes. Although the biasing effect of attitudes was notable, people’s cognitive sophistication lessened it and helped people to notice weaknesses in the poorly justified arguments. Specifically, those with high analytic thinking and scientific literacy were slightly better than others at noticing fallacies when the fallacy claims went against their attitudes, and moderately better at it when their attitudes aligned with the fallacies’ conclusions. Good scientific reasoning skills and high actively open-minded thinking were the aspects of cognitive sophistication that most notably increased with correct fallacy detection.

Much remains still to be learned about the common processes as well as individual differences that underlie the way people use, process, and evaluate everyday arguments. However, investigating real-world argumentation is challenging due to its contextual and knowledge-dependent nature. We believe that systematizing argument studies would help approach this critical topic and hope that, together with previous studies, this study inspires and aids future investigations into people’s ability to notice or willingness to care about the credibility of justifications.

## Electronic supplementary material

Below is the link to the electronic supplementary material.

Supplementary file1 (DOCX 608 KB)

## Data Availability

Not applicable.

## Appendix

**Table 5:** Fallacies in the Study

Name	Content
ProCan_Adhom	Cannabis should be legalized because people who oppose it create unnecessary horror scenarios of mild drugs in their mind.
ProEut_Adhom	Euthanasia should be legalized because people who oppose it cannot put themselves in the sufferers’ shoes.
ProGmo_ Adhom	Genetic modification of food should be allowed because people who oppose it do not care about the wellbeing of mankind.
ProImmi_Adhom	Immigration to Finland should not be made stricter because people who oppose immigration only think about themselves.
ProNucl_Adhom	Nuclear power should be increased in the world because people who oppose it do not care about future generations' quality of life.
ConCan_Adhom	Cannabis should not be legalized because people who support it have too rosy view of drugs.
ConEut_Adhom	Euthanasia should not be legalized because people who support it do not understand the importance of life.
ConGmo_Adhom	Genetic modification of food should not be allowed because people who support it do not care about the livelihood of small farmers.
ConImmi_Adhom	Immigration to Finland should be made stricter because people who support immigration do not care about the best interest of native Finns.
ConNucl_Adhom	Nuclear power should not be increased in the world because people who support it do not care about the catastrophic consequences of serious nuclear accidents.
ProCan_Natur	Cannabis should be legalized because cannabis is a clean natural product.
ProEut_Natur	Euthanasia should be legalized because it is natural to help a suffering person.
ProGmo_Natur	Genetic modification of food should be allowed because genetic mutations happen in nature anyway.
ProImmi_Natur	Immigration to Finland should not be made stricter because it is a natural part of the present-day world that people move across country borders.
ProNucl_Natur	Nuclear power should be increased in the world because the sun also produces radiation energy with similar principles.
ConCan_Natur	Cannabis should not be legalized because cannabis is similar to synthetic chemicals.
ConEut_Natur	Euthanasia should not be legalized because it is not a natural way to die.
ConGmo_Natur	Genetic modification of food should not be allowed because people should not get involved with natural cycles.
ConImmi_Natur	Immigration to Finland should be made stricter because mixing cultures in this scale is not natural.
ConNucl_Natur	Nuclear power should not be increased in the world because forcefully breaking atomic nuclei is unnatural.
ProCan_Circul	Cannabis should be legalized because legalizing lowers crime.
ProEut_Circul	Euthanasia should be legalized because the right to die belongs to those who suffer.
ProGmo_Circul	Genetic modification of food should be allowed because it provides new kinds of food to people.
ProImmi_Circul	Immigration to Finland should not be made stricter because more people would then have to stay in their home countries.
ProNucl_Circul	Nuclear power should be increased in the world because then a larger portion of energy would come from nuclear power.
ConCan_Circul	Cannabis should not be legalized because it makes an illegal thing legal.
ConEut_Circul	Euthanasia should not be legalized because killing is wrong.
ConGmo_Circul	Genetic modification of food should not be allowed because it requires the modification of original genes.
ConImmi_Circul	Immigration to Finland should be made stricter because it increases the amount of crimes conducted by immigrants.
ConNucl_Circul	Nuclear power should not be increased in the world because then the number of nuclear power plants would increase.
ProCan_Ignor	Cannabis should be legalized because it is unknown that its legalizing would have adverse effects on the public’s health.
ProEut_Ignor	Euthanasia should be legalized because it is unknown that legalizing it would have harmful consequences.
ProGmo_Ignor	Genetic modification of food should be allowed because it is unknown that it would contain risks.
ProImmi_Ignor	Immigration to Finland should not be made stricter because the benefits of immigration will not be revealed until later.
ProNucl_Ignor	Nuclear power should be increased in the world because the technology used in nuclear power could be completely safe in the future.
ConCan_Ignor	Cannabis should not be legalized because not all consequences of legalizing are known.
ConEut_Ignor	Euthanasia should not be legalized because it is unknown which illnesses will have a cure in the future.
ConGmo_Ignor	Genetic modification of food should not be allowed because the health risks that follow from it are unknown.
ConImmi_Ignor	Immigration to Finland should be made stricter because the way immigrants affect Finnish society is unknown.
ConNucl_Ignor	Nuclear power should not be increased in the world because unpredictable events make it risky.
ProCan_Slippery	Cannabis should be legalized because then many users of harder drugs would start using it instead.
ProEut_Slippery	Euthanasia should be legalized because then fewer and fewer people would commit suicide.
ProGmo_Slippery	Genetic modification of food should be allowed because it would remove famine in the world.
ProImmi_Slippery	Immigration to Finland should not be made stricter because it would lead to a lower appreciation of human rights.
ProNucl_Slippery	Nuclear power should be increased in the world because otherwise the development of effective and clean fusion energy will be abandoned.
ConCan_Slippery	Cannabis should not be legalized because cannabis use increases the use of hard drugs.
ConEut_Slippery	Euthanasia should not be legalized because with time its use would be expanded to less sick patients.
ConGmo_Slippery	Genetic modification of food should not be allowed because it leads to the selling of groceries that contain health risks.
ConImmi_Slippery	Immigration to Finland should be made stricter because immigration lowers the effort put into the homeland’s own problems.
ConNucl_Slippery	Nuclear power should not be increased in the world because it would lead to the spread of nuclear weapons into new countries.

The names include information about the topic (Can = cannabis, Eut = euthanasia, Gmo = genetic modification of food, Immi = immigration, Nucl = nuclear power), the side of the topic (pro or con), and the fallacy type (Adhom = ad hominem, Natur = appeal to nature, Circul = circular reasoning, Ignor = appeal to ignorance, Slippery = slippery slope arguments).

see Table 5

## Footnotes

1 We use the term *motivated sophistication* to refer to the idea that cognitive sophistication (i.e., high analytic thinking styles, scientific literacy, and intelligence) increases mysided thinking, also referred to as Motivated System 2 Reasoning (e.g., Pennycook & Rand, 2021b) or identity-protective cognition (e.g., Kahan et al., 2017). This is different from people’s general tendency to be mysided thinkers, often referred to as motivated reasoning (e.g., Kunda, 1990).

## Declarations

### Open practices statement

The data that support the findings of this study are openly available on Open Science Framework at https://osf.io/hc4qm/?view_only=15ed9dc592c649a1841651d42151274d, Susceptibility to Poor Arguments. This study was not preregistered.
