# Supplementary material for: Susceptibility to poor arguments: The interplay of cognitive sophistication and attitudes
Source: Mem Cognit. 2024 Apr 24;52(7):1579–96. doi: 10.3758/s13421-024-01564-1 (PMC11522166; doi:10.3758/s13421-024-01564-1)
Supplement: Supplementary file 1 — Supplementary file1 (DOCX 608 KB) [file 13421_2024_1564_MOESM1_ESM.docx]

**Supplemental Materials**

**Table S1**

*Correlations Between the Fallacy Types Overall and per Topic Sides*

|  |  |  | All | | | | |  | Pro topic | | | | |  | Con topic | | | | |  | |
| --- | --- | --- | --- | --- | --- | --- | --- | --- | --- | --- | --- | --- | --- | --- | --- | --- | --- | --- | --- | --- | --- |
|  | M | SD | Adhom | Natur | Circul | Ignor | Slipp |  | Adhom | Natur | Circul | Ignor | Slipp |  | Adhom | Natur | Circul | Ignor | Slipp | All pro |  |
| All |  |  |  |  |  |  |  |  |  |  |  |  |  |  |  |  |  |  |  |  |  |
| Adhom | 1.71 | 0.59 |  |  |  |  |  |  |  |  |  |  |  |  |  |  |  |  |  |  |  |
| Natur | 2.19 | 0.55 | .56 |  |  |  |  |  |  |  |  |  |  |  |  |  |  |  |  |  |  |
| Circul | 2.41 | 0.45 | .48 | .54 |  |  |  |  |  |  |  |  |  |  |  |  |  |  |  |  |  |
| Ignor | 2.61 | 0.47 | .30 | .39 | .38 |  |  |  |  |  |  |  |  |  |  |  |  |  |  |  |  |
| Slipp | 2.67 | 0.52 | .25 | .33 | .32 | .36 |  |  |  |  |  |  |  |  |  |  |  |  |  |  |  |
| Pro |  |  |  |  |  |  |  |  |  |  |  |  |  |  |  |  |  |  |  |  |  |
| Adhom | 1.65 | 0.61 | .90 | .49 | .43 | .28 | .20 |  |  |  |  |  |  |  |  |  |  |  |  |  |  |
| Natur | 2.43 | 0.65 | .42 | .78 | .43 | .30 | .22 |  | .47 |  |  |  |  |  |  |  |  |  |  |  |  |
| Circul | 2.77 | 0.58 | .20 | .21 | .67 | .23 | .19 |  | .30 | .40 |  |  |  |  |  |  |  |  |  |  |  |
| Ignor | 2.35 | 0.65 | .24 | .26 | .28 | .73 | .21 |  | .32 | .36 | .36 |  |  |  |  |  |  |  |  |  |  |
| Slipp | 2.73 | 0.63 | .12 | .18 | .22 | .27 | .73 |  | .19 | .30 | .38 | .33 |  |  |  |  |  |  |  |  |  |
| Con |  |  |  |  |  |  |  |  |  |  |  |  |  |  |  |  |  |  |  |  |  |
| Adhom | 1.78 | 0.70 | .93 | .53 | .44 | .29 | .26 |  | .71 | .33 | .10 | .16 | .06 |  |  |  |  |  |  |  |  |
| Natur | 1.94 | 0.70 | .47 | .82 | .44 | .32 | .31 |  | .33 | .33 | -.01 | .09 | .01 |  | .51 |  |  |  |  |  |  |
| Circul | 2.06 | 0.66 | .47 | .55 | .77 | .31 | .27 |  | .34 | .27 | .09 | .10 | <.01 |  | .50 | .61 |  |  |  |  |  |
| Ignor | 2.88 | 0.62 | .20 | .30 | .26 | .71 | .31 |  | .10 | .08 | -.02 | .07 | .07 |  | .26 | .38 | .35 |  |  |  |  |
| Slipp | 2.61 | 0.70 | .24 | .32 | .27 | .28 | .79 |  | .11 | .06 | -.04 | .03 | .21 |  | .32 | .43 | .39 | .39 |  |  |  |
| All pro | 2.39 | 0.44 | .52 | .55 | .59 | .53 | .45 |  | .63 | .73 | .69 | .68 | .64 |  | .37 | .22 | .24 | .09 | .11 |  |  |
| All con | 2.25 | 0.51 | .61 | .69 | .59 | .52 | .54 |  | .42 | .30 | .05 | .13 | .11 |  | .69 | .81 | .77 | .64 | .70 | .29 |  |

*Note.* *r*s ≥ |.10|: p < .001; *r*s ≥ |.08|: p < .01; *r*s ≥ |.06|: p < .05. Abbreviations: Adhom = ad hominem, Natur = appeal to naturalness, Circul = circular reasoning, Ignor = appeal to ignorance, Slipp = slippery slope arguments, Pro = arguments arguing for a topic, Con = arguments arguing against a topic. All mean differences between the pro and con topic fallacies were significant at p ≤ .001 after Bonferroni correction except for pro slipp vs. con slipp (p < .01), pro circul vs. con ignor (p < .05), and pro slipp vs. pro circul (p > .05).

**Table S2**

*Correlations Between the Cognitive Characteristics, Fallacy Types, and Attitudes*

|  | Familiarity | COGSOP | FI | NfC | CRT | IH | AOT | SRS |
| --- | --- | --- | --- | --- | --- | --- | --- | --- |
| COGSOP | .20 |  |  |  |  |  |  |  |
| FI | -.13 | -.61 |  |  |  |  |  |  |
| NfC | .33 | .37 | -.18 |  |  |  |  |  |
| CRT | .09 | .66 | -.15 | .23 |  |  |  |  |
| IH | .21 | .21 | -.09 | .32 | .05 |  |  |  |
| AOT | .18 | .68 | -.28 | .31 | .25 | .36 |  |  |
| SRS | .15 | .70 | -.20 | .28 | .38 | .08 | .34 |  |
| Adhom All | -.13 | -.51 | .22 | -.23 | -.32 | -.14 | -.40 | -.41 |
| Adhom Pro | -.11 | -.41 | .16 | -.21 | -.28 | -.12 | -.31 | -.36 |
| Adhom Con | -.13 | -.50 | .23 | -.22 | -.30 | -.14 | -.43 | -.39 |
| Natural All | -.18 | -.53 | .30 | -.26 | -.28 | -.11 | -.43 | -.41 |
| Natural Pro | -.13 | -.32 | .18 | -.16 | -.16 | -.02 | -.22 | -.29 |
| Natural Con | -.16 | -.52 | .29 | -.27 | -.27 | -.15 | -.46 | -.37 |
| Circular All | -.07 | -.35 | .17 | -.15 | -.22 | -.07 | -.25 | -.30 |
| Circular Pro | <.01 | <.01 | .01 | -.02 | .01 | .04 | .06 | -.05 |
| Circular Con | -.08 | -.45 | .20 | -.18 | -.28 | -.11 | -.39 | -.35 |
| Ignorance All | -.06 | -.22 | .16 | -.11 | -.15 | -.04 | -.15 | -.13 |
| Ignorance Pro | -.03 | -.13 | .08 | -.09 | -.09 | -.02 | -.07 | -.09 |
| Ignorance Con | -.07 | -.18 | .15 | -.09 | -.12 | -.05 | -.13 | -.10 |
| Slippery All | -.09 | -.18 | .14 | -.11 | -.06 | -.02 | -.14 | -.13 |
| Slippery Pro | -.05 | .03 | .04 | -.03 | .07 | .05 | .05 | <.01 |
| Slippery Con | -.09 | -.26 | .15 | -.13 | -.14 | -.07 | -.23 | -.18 |
| All Pro | -.10 | -.23 | .13 | -.14 | -.12 | -.02 | -.14 | -.22 |
| All Con | -.14 | -.51 | .28 | -.24 | -.29 | -.13 | -.43 | -.37 |
| Attitude Can | .13 | .34 | -.14 | .14 | .21 | .15 | .35 | .19 |
| Attitude Eut | -.08 | .11 | -.05 | .03 | .05 | .07 | .18 | .01 |
| Attitude GMO | .11 | .46 | -.27 | .16 | .31 | .10 | .34 | .33 |
| Attitude Immig | .13 | .15 | -.04 | .05 | .02 | .06 | .21 | .12 |
| Attitude Nucl | .08 | .31 | -.20 | .18 | .22 | .06 | .17 | .23 |

*Note.* *r*s ≥ |.10|: p < .001; *r*s ≥ |.08|: p < .01; *r*s ≥ |.06|: p < .05. Abbreviations: Familiarity = familiarity with argumentation, COGSOP = cognitive sophistication, FI = faith in intuition, NfC = need for cognition, CRT = cognitive reflection test, IH = intellectual humility, AOT = actively open-minded thinking,

SRS = scientific reasoning skills, Adhom = ad hominem, Natural = appeal to naturalness, Circular = circular reasoning, Ignorance = appeal to ignorance, Slippery = slippery slope arguments, Pro = arguments arguing for a topic, Con = arguments arguing against a topic, Can = Cannabis, Eut = Euthanasia, GMO = Genetically modified food, Immig = Immigration, Nucl = Nuclear power.

**Table S3**

|  | *Cannabis+* | *Cannabis-* | *Euthanasia+* | *Euthanasia-* | *GMO+* | *GMO-* | *Immigration+* | *Immigration-* | *Nuclear+* | *Nuclear-* |
| --- | --- | --- | --- | --- | --- | --- | --- | --- | --- | --- |
| Block 3 |  |  |  |  |  |  |  |  |  |  |
| Age | .01 | .03 | .04 | .03 | .07** | -.03 | .01 | -.03 | .02 | .06* |
| Familiarity | -.03 | -.04 | <.01 | .02 | .01 | -.04 | **-.08**** | .03 | -.03 | -.02 |
| Attitudeᶧ | **.46***** | **.53***** | **.41***** | **.46***** | **.50***** | **.48***** | **.40***** | **.50***** | **.29***** | **.40***** |
| FI | .05* | **.07**** | **.09***** | .06* | .07** | **.07***** | **.08**** | .06* | **.08**** | **.08**** |
| NfC | -.03 | -.06* | -.02 | <.01 | <.01 | -.02 | -.07* | -.01 | -.06 | -.04 |
| CRT | -.04 | -.04 | -.07** | .01 | -.06* | -.04 | -.02 | -.05* | -.06* | **-.09***** |
| IH | **.08**** | .02 | .04 | .03 | .03 | .02 | **.08**** | -.02 | .06* | .04 |
| AOT | **-.12***** | -.06* | **-.09**** | **-.14***** | -.07* | **-.13***** | **-.10**** | **-.13***** | **-.27***** | **-.09***** |
| SRS | **-.16***** | -.06** | **-.17***** | -.03 | **-.13***** | **-.16***** | **-.12***** | -.05 | **-.17***** | **-.14***** |
| Att*FI | .05 | .01 | .05* | .04 | .01 | -.01 | .01 | .06** | **.08**** | .02 |
| Att*NfC | .07* | -.01 | .03 | .05 | .02 | .06* | .06* | .01 | .04 | -.02 |
| Att*CRT | -.08** | **-.09***** | -.07* | -.07** | **-.10***** | -.04 | -.03 | **-.08**** | -.06* | -.06* |
| Att*IH | -.02 | .02 | .04 | .01 | <.01 | -.01 | -.04 | -.04 | .02 | .02 |
| Att*AOT | -.03 | -.04 | -.04 | -.04 | -.07* | -.01 | -.07* | -.04 | -.07* | -.06* |
| Att*SRS | **-.12***** | **-.07**** | **-.08**** | **-.10***** | -.02 | -.06* | -.08** | **-.09***** | **-.09***** | -.07** |
| *Δ Adj. R^2^* | *8.4%* | *4.8%* | *9.7%* | *4.7%* | *4.5%* | *6.9%* | *5.6%* | *8.7%* | *18.1%* | *8.8%* |

*Betas of Blocks 3 in Hierarchical Regression Analyses With All Cognitive Characteristics Predicting the Acceptance of Pro and Con Topic Fallacious Arguments*

*Note.* ****p* < .001; ***p* < .01; **p* < .05. Betas significant at *p* < .05 after Bonferroni correction are bolded. Blocks 1 and 2 were identical to Table 3 and controlled the effects of age, familiarity with argumentation, and attitudes. For each topic, the plus sign (+) denotes pro topic fallacious arguments and the minus sign (-) denotes con topic fallacious arguments. ᶧAttitude toward a topic always aligns with the argument claims, ranging from negative to positive for the pro topic fallacies, and from positive to negative for the con topic fallacies. Abbreviations: Familiarity = familiarity with argumentation, FI = faith in intuition, NfC = need for cognition, CRT = cognitive reflection test, IH = intellectual humility, AOT = actively open-minded thinking, SRS = scientific reasoning skills, Att = attitude, * = interaction terms. Changes in Adj. R^2^ ≥ 0.8 % were significant at p < .05 after Bonferroni correction.

**Table S4**

|  | *Cannabis+* | *Cannabis-* | *Euthanasia+* | *Euthanasia-* | *GMO+* | *GMO-* | *Immigration+* | *Immigration-* | *Nuclear+* | *Nuclear-* |
| --- | --- | --- | --- | --- | --- | --- | --- | --- | --- | --- |
| Block 3 |  |  |  |  |  |  |  |  |  |  |
| Age | -.04 ─ .07 | -.02 ─ .07 | -.006 ─ .10 | -.02 ─ .07 | .02 ─ .13 | -.07 ─ .02 | -.05 ─ .06 | -.07 ─ .02 | -.03 ─ .07 | .009 ─ .10 |
| Familiarity | -.08 ─ .02 | -.08 ─ .01 | -.05 ─ .05 | -.03 ─ .06 | -.04 ─ .06 | -.08 ─ .002 | -.14 ─ -.03 | -.01 ─ .08 | -.08 ─ .02 | -.07 ─ .03 |
| Attitudeᶧ | .40 ─ .51 | .48 ─ .57 | .36 ─ .46 | .41 ─ .50 | .44 ─ .55 | .44 ─ .53 | .35 ─ .45 | .45 ─ .54 | .24 ─ .34 | .35 ─ .45 |
| FI | .002 ─ .10 | .02 ─ .11 | .04 ─ .14 | .01 ─ .11 | .02 ─ .13 | .03 ─ .12 | .03 ─ .13 | .01 ─ .10 | .03 ─ .13 | .03 ─ .12 |
| NfC | -.08 ─ .03 | -.10 ─ -.007 | -.07 ─ .04 | -.05 ─ .05 | -.06 ─ .05 | -.07 ─ .03 | -.13 ─ -.02 | -.06 ─ .04 | -.11 ─ .001 | -.09 ─ .007 |
| CRT | -.10 ─ .02 | -.09 ─ .004 | -.13 ─ -.02 | -.04 ─ .06 | -.12 ─ -.003 | -.09 ─ .003 | -.08 ─ .04 | -.10 ─ -.001 | -.11 ─ -.003 | -.14 ─ -.04 |
| IH | .03 ─ .13 | -.03 ─ .06 | -.02 ─ .09 | -.02 ─ .08 | -.03 ─ .08 | -.02 ─ .07 | .03 ─ .14 | -.07 ─ .02 | .003 ─ .11 | -.009 ─ .09 |
| AOT | -.18 ─ -.06 | -.12 ─ -.01 | -.15 ─ -.03 | -.20 ─ -.09 | -.13 ─ -.01 | -.17 ─ -.08 | -.16 ─ -.04 | -.18 ─ -.08 | -.32 ─ -.21 | -.14 ─ -.04 |
| SRS | -.22 ─ -.11 | -.11 ─ -.02 | -.23 ─ -.12 | -.08 ─ .03 | -.19 ─ -.07 | -.21 ─ -.11 | -.18 ─ -.06 | -.10 ─ .002 | -.23 ─ -.11 | -.19 ─ -.09 |
| Att*FI | -.005 ─ .10 | -.03 ─ .05 | .005 ─ .10 | -.01 ─ .08 | -.05 ─ .06 | -.05 ─ .04 | -.05 ─ .06 | .02 ─ .11 | .03 ─ .13 | -.02 ─ .07 |
| Att*NfC | .01 ─ .12 | -.05 ─ .04 | -.03 ─ .08 | -.002 ─ .10 | -.03 ─ .08 | .01 ─ .10 | .003 ─ .11 | -.04 ─ .05 | -.01 ─ .10 | -.06 ─ .03 |
| Att*CRT | -.13 ─ -.02 | -.13 ─ -.04 | -.12 ─ -.01 | -.13 ─ -.02 | -.16 ─ -.05 | -.09 ─ .002 | -.09 ─ .03 | -.13 ─ -.03 | -.11 ─ -.009 | -.11 ─ -.01 |
| Att*IH | -.07 ─ .04 | -.03 ─ .07 | -.02 ─ .09 | -.05 ─ .06 | -.05 ─.06 | -.05 ─ .04 | -.10 ─ .02 | -.08 ─ .01 | -.03 ─ .07 | -.03 ─ .06 |
| Att*AOT | -.09 ─ .03 | -.09 ─ .02 | -.10 ─ .02 | -.09 ─ .02 | -.13 ─ -.004 | -.06 ─ .05 | -.13 ─ -.005 | -.10 ─ .01 | -.13 ─ -.01 | -.11 ─ -.003 |
| Att*SRS | -.19 ─ -.07 | -.12 ─ -.02 | -.14 ─ -.03 | -.15 ─ -.05 | -.08 ─ .04 | -.11 ─ -.01 | -.14 ─ -.02 | -.15 ─ -.05 | -.15 ─ -.04 | -.13 ─ -.02 |

*Confidence Intervals (95 %) of the Betas in Blocks 3 in the Hierarchical Regression Analyses With All Cognitive Characteristics*

*Note.* Confidence intervals for Blocks 1 and 2 are shown in Table S4. For each topic, the plus sign (+) denotes pro topic fallacious arguments and the minus sign (-) denotes con topic fallacious arguments. ᶧAttitude toward a topic always aligns with the argument claims, ranging from negative to positive for pro topic fallacies, and positive to negative for con topic fallacies. Abbreviations: Familiarity = familiarity with argumentation, FI = faith in intuition, NfC = need for cognition, CRT = cognitive reflection test, IH = intellectual humility, AOT = actively open-minded thinking, SRS = scientific reasoning skills, Att = attitude, * = interaction terms.

**Figure S1**

*Correlations Between Fallacy Acceptance and the Cognitive Characteristics That Showed Significant Interactions With Attitudes (p < .005)*

*in the Regression Analyses, Calculated Separately for Participants Whose Attitudes Aligned With and Opposed the Fallacy Claims.*

*
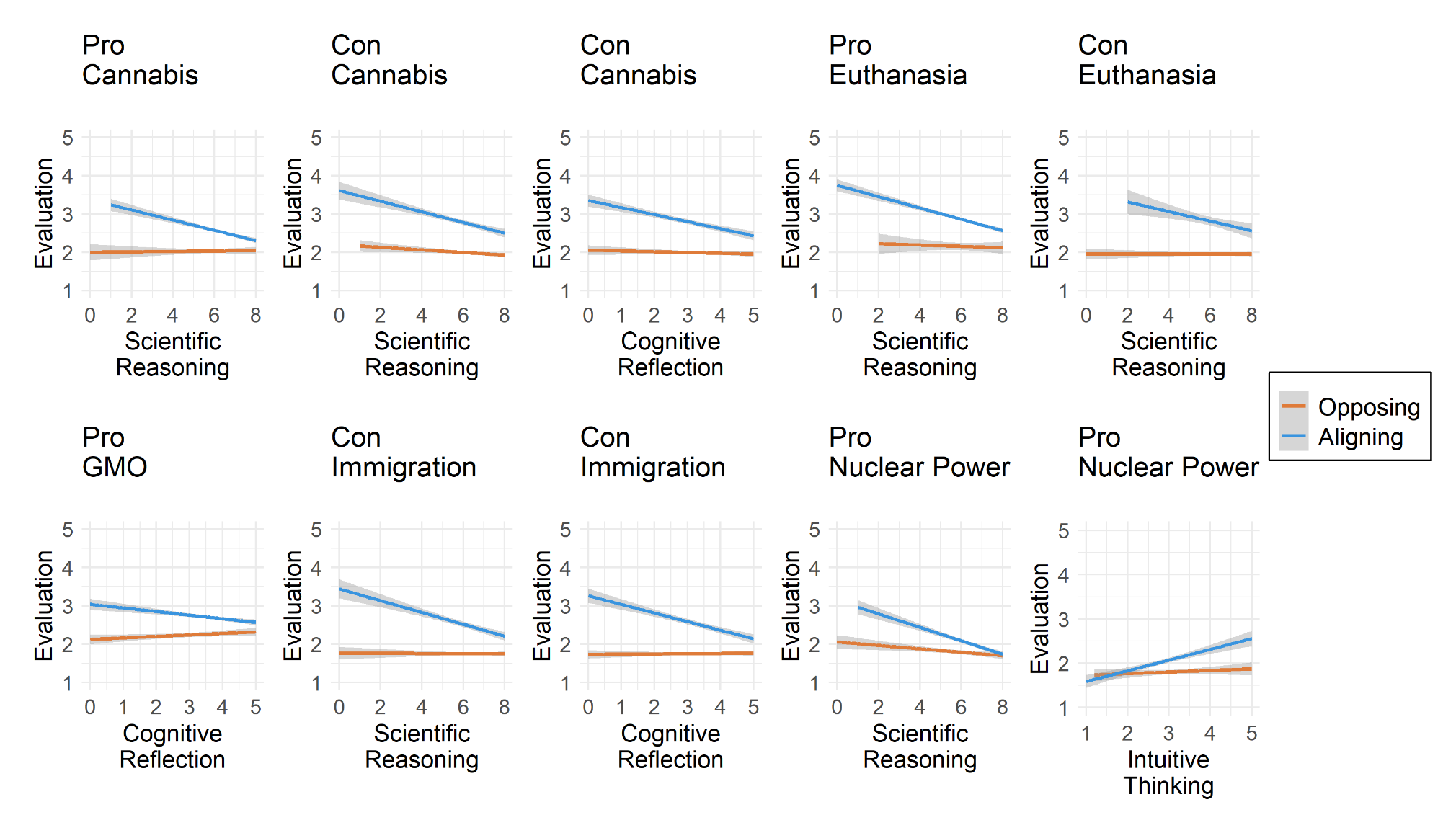
*

*Note.* The weak positive correlation (r = . 09) between cognitive reflection and acceptance of attitude-opposing Pro GMO fallacies was not significant at p < .05. All correlations between the cognitive characteristics and acceptance of aligning fallacies were significant at p < .001.

**Table S5**

*Confidence Intervals (95 %) of the Betas in the Hierarchical Regression Analyses With Cognitive Sophistication*

|  | *Cannabis+* | | *Cannabis-* | | *Euthanasia+* | | *Euthanasia-* | | *GMO+* | | *GMO-* | | *Immigration+* | | *Immigration-* | | *Nuclear+* | | *Nuclear-* | |  |
| --- | --- | --- | --- | --- | --- | --- | --- | --- | --- | --- | --- | --- | --- | --- | --- | --- | --- | --- | --- | --- | --- |
| Block 1 |  | |  | |  | |  | |  | |  | |  | |  | |  | |  | |  |
| Age | -.11 ─ .02 | | .16 ─ .26 | | .03 ─ 14 | | .03 ─ .14 | | -.09 ─ .02 | | .15 ─ .25 | | .04 ─ .15 | | -.09 ─ .02 | | -.01 ─ .10 | | .17 ─ .27 | |  |
| Familiarity | -.08 ─ .03 | | -.20 ─ -.09 | | -.16 ─ -.05 | | -.02 ─ .09 | | -.04 ─ .07 | | -.20 ─ -.09 | | -.13 ─ -.02 | | -.15 ─ -.04 | | -.16 ─ -.05 | | -.16 ─ -.05 | |  |
| Block 2 |  | |  | |  | |  | |  | |  | |  | |  | |  | |  | |  |
| Age | -.01 ─ .09 | | .03 ─ .12 | | .07 ─ .17 | | -.004 ─ .09 | | .04 ─ .15 | | -.04 ─ .06 | | .004 ─ .11 | | -.02 ─ .07 | | .03 ─ .14 | | .07 ─ .16 | |  |
| Familiarity | -.12 ─ -.02 | | -.11 ─ -.03 | | -.12 ─ -.02 | | -.05 ─ .04 | | -.07 ─ .03 | | -.13 ─ -.05 | | -.18 ─ -.08 | | -.06 ─ .03 | | -.17 ─ -.06 | | -.13 ─ -.03 | |  |
| Attitudeᶧ | .36 ─ .46 | | .54 ─ .63 | | .36 ─ .46 | | .47 ─ .57 | | .37 ─ .47 | | .56 ─ .65 | | .33 ─ .43 | | .52 ─ .61 | | .15 ─ .26 | | .42 ─ .52 | |  |
| Block 3 |  | |  | |  | |  | |  | |  | |  | |  | |  | |  | |  |
| Age | | -.04 ─ .06 | | -.02 ─ .07 | | -.01 ─ .09 | | -.04 ─ .05 | | .02 ─ .12 | | -.08 ─ .01 | | -.06 ─ .05 | | -.08 ─ .009 | | -.05 ─ .05 | | .01 ─ .10 | |
| Familiarity | | -.08 ─ .02 | | -.09 ─ -.01 | | -.05 ─ .05 | | -.03 ─ .06 | | -.04 ─ .06 | | -.09 ─ -.005 | | -.14 ─ -.04 | | -.02 ─ .06 | | -.10 ─ .002 | | -.07 ─ .02 | |
| Attitudeᶧ | | .41 ─ .51 | | .48 ─ .56 | | .37 ─ .47 | | .42 ─ .52 | | .44 ─ .55 | | .44 ─ .53 | | .36 ─ .46 | | .46 ─ .55 | | .24 ─ .35 | | .36 ─ .45 | |
| Cognition | | -.30 ─ -.20 | | -.22 ─ -.13 | | -.34 ─ -.24 | | -.19 ─ -.10 | | -.27 ─ -.16 | | -.32 ─ -.23 | | -.27 ─ -.17 | | -.25 ─ -.15 | | -.45 ─ -.35 | | -.32 ─ -.23 | |
| Att*Cognition | | -.22 ─ -.12 | | -.18 ─ -.10 | | -.19 ─ -.10 | | -.20 ─ -.10 | | -.17 ─ -.07 | | -.10 ─ -.01 | | -.17 ─ -.07 | | -.24 ─ -.16 | | -.23 ─ -.13 | | -.19 ─ -.10 | |

*Note.* For each topic, the plus sign (+) denotes pro topic fallacious arguments and the minus sign (-) denotes con topic fallacious arguments. ᶧAttitude toward a topic always aligns with the argument claims, ranging from negative to positive for pro topic fallacies, and positive to negative for con topic fallacies. Abbreviations: Familiarity = familiarity with argumentation, Cognition = cognitive sophistication, Att*Cognition = interaction between attitude and cognitive sophistication.
